# Loss of Stromal Caveolin-1 Expression: A Novel Tumor Microenvironment Biomarker That Can Predict Poor Clinical Outcomes for Pancreatic Cancer

**DOI:** 10.1371/journal.pone.0097239

**Published:** 2014-06-20

**Authors:** Tao Shan, Hongwei Lu, Hong Ji, Yiming Li, Jian Guo, Xi Chen, Tao Wu

**Affiliations:** 1 Department of General Surgery, Second Affiliated Hospital of Medical College, Xi’an Jiaotong University, Xi’an, Shaanxi, China; 2 Department of Hepatobiliary Surgery, First Affiliated Hospital of Medical College, Xi’an Jiaotong University, Xi’an, Shaanxi, China; IPMC, CNRS UMR 7275 UNS, France

## Abstract

**Aims:**

Cancer development and progression is not only associated with the tumor cell proliferation but also depends on the interaction between tumor cells and the stromal microenvironment. A new understanding of the role of the tumor microenvironment suggests that the loss of stromal caveolin-1 (Cav-1) as a key regulator may become a potential therapy target. This study aims to elucidate whether stromal Cav-1 expression in pancreatic cancer can be a strong prognosis biomarker.

**Methods:**

Tissue samples from 45 pancreatic cancer patients were studied. Parenchyma and stroma were separated and purified using laser capture microdissection. Stromal Cav-1 expression was measured from pancreatic cancer, paraneoplastic, and normal tissue using immunohistochemistry. We analyzed the correlation of stromal Cav-1 expression with clinicopathologic features and prognostic indicators, such as tumor marker HER-2/neu gene.

**Results:**

Specimens from six patients (13.3%) showed high levels of stromal Cav-1 staining, those from eight patients (17.8%) showed a lower, intermediate level of staining, whereas those from 31 patients (68.9%) showed an absence of staining. Cav-1 expression in cancer-associated fibroblasts was lower than that in paracancer-associated and in normal fibroblasts. Stromal Cav-1 loss was associated with TNM stage (*P = *0.018), lymph node metastasis (*P* = 0.014), distant metastasis (*P* = 0.027), and HER-2/neu amplification (*P* = 0.007). The relationships of age, sex, histological grade, and tumor size with stromal Cav-1 expression were not significant (*P*>0.05). A negative correlation was found between circulating tumor cells and stromal Cav-1 expression (*P*<0.05).

**Conclusion:**

The loss of stromal Cav-1 in pancreatic cancer was an independent prognostic indicator, thus suggesting that stromal Cav-1 may be an effective therapeutic target for patients with pancreatic cancer.

## Introduction

Pancreatic cancer has a survival rate of less than 25% at five years after partial pancreaticoduodenectomy and is one of the most aggressive and intractable human malignant tumors [1]. Recent studies have shown that the microenvironment serve a function in the progression of malignant epithelial tumors, thus highlighting the importance of understanding of stromal cells in the prevention of cancer cell aggression and anti-cancer treatment strategies [2]. To understand fully the mechanism driving tumor recurrence, metastasis, and clinical outcome in cancer patients, the role of the tumor microenvironment must be examined. In particular, cancer-associated fibroblasts (CAFs) have been found to serve a crucial function through paracrine interactions with adjacent epithelial cancer cells [3], [4].

Caveolins (Cav) comprise a family of scaffolding proteins that coat 50 nm to 100 nm plasma membrane invaginations [5]. The Cav family is composed of three isoforms: Cav-1, Cav-2, and Cav-3. The Cav-1 gene is located in chromosome 7 (locus 7q31.1) and includes three exons (30, 165, and 342 bp) and two introns (1.5 and 32 kb). Cav-1 is a structural component of caveolae involved in diverse cellular functions, such as vesicular transport, cholesterol homeostasis, and signal transduction [6]. Despite a growing body of evidence on Cav-1 implication in tumorigenesis, whether Cav-1 serves as a tumor suppressor or as an oncogene remains unclear. These contradictory results are probably derived from the study of malignant epithelial tumors [7], [8], [9]. A new paradigm of “the autophagic tumor stroma model of cancer metabolism” was recently introduced to facilitate understanding of the function of tumor microenvironments [10], [11]. In this model, the loss of stromal Cav-1 as a key regulator is a potential therapy target, thus suggesting the prognostic importance of stromal Cav-1 [12]. Loss of stromal Cav-1 is the single independent predictor of early breast cancer recurrence and progression [7]. Ayala et al. reported that loss of stromal Cav-1 contributed to the metastatic behavior of prostate cancer cells through a mechanism that involving the upregulation of TGF-β1 and SNCG through Akt activation [13]. Zhao et al. reported that Cav-1 expression level in CAFs predicted gastric cancer outcomes [14]. Karen et al. stated that the loss of stromal Cav-1 expression in malignant melanoma metastases predicted poor survival [15]. However, stromal Cav-1 expression in pancreatic cancer, as well as its clinical significance, remains unclear. To elucidate the function of stromal Cav-1 in pancreatic cancer, we investigated the stromal Cav-1 expression in pancreatic cancer specimens, along with the correlation of stromal Cav-1 expression with tumor marker HER-2/neu and a number of circulating tumor cells (CTCs).

## Materials and Methods

### Patient Specimens

From January 2007 to December 2012, pancreatic cancer tissues (including adequately sized tumor tissue samples and tissue samples obtained from areas within 2.0 cm of the tumor) were obtained from 45 patients undergoing partial pancreaticoduodenectomy (Whipple resection) for pancreatic cancer at the Department of Hepatobiliary and Pancreatic Surgery, First and Second Affiliated Hospitals of Xi’an Jiaotong University. A portion of each specimen was frozen and prepared for laser capture microdissection (LCM); other specimen portions were fixed with 10% formalin for histological studies. Ten samples containing normal pancreatic tissues from patients who received partial pancreatectomy for benign tumors were used as normal controls. Of the 45 study patients, 24 were men, and 21 were women. The median age at the time of surgery was 64.5 years (range from 44 years to 82 years). All 45 patients had pancreatic ductal adenocarcinoma. Tumor stage and histopathological grading were recorded according to the classification of the International Union Against Cancer. Three patients had stage I tumors, 11 had stage II, 27 had stage III, and 4 patients had stage IV tumors. Histological tumor grades were as follows: 7 patients had grade I, 20 had grade II, and 18 had grade III tumors. All patients participated in the follow-up procedure. The median time for follow-up was 22 months (range from 4 months to 52 months). These samples were obtained from donors or the next of kin who signed a written informed consent. The studies were approved by the Institutional Review Board and Ethics Committee of Xi’an Jiaotong University, China.

### Immunohistochemistry

Cav-1 protein was detected by immunohistochemistry using the standardized streptavidin-peroxidase method. Tissue sections (4 µm) were incubated overnight with a standard primary antibody concentration. The slides were incubated for 30 min with biotinylated goat anti-rabbit IgG, followed by incubation with peroxidase-conjugated streptavidin for 20 min at room temperature. Color was developed using 0.02% solution of 3, 3′-diaminobenzidine in 50 mM Tris–HCl buffer (pH 7.6) for 5 min to 7 min. Finally, the sections were counterstained with hematoxylin, rinsed with water, dehydrated, cleared, and cover slipped. In negative controls for immunostaining, the primary antibody was replaced with non-immune goat or rabbit serum. The number of stained cells per 1000 was determined under a microscope (Olympus Optical Co, Ltd, Tokyo, Japan) in three visual fields, at a magnification of ×400. When the total number of cells observed under the microscope was less than 1000, all cells were counted. Staining was scored semiquantitatively as negative (0; no staining), weak (1; either diffuse weak staining or strong staining in less than 30% of stromal cells), or strong (2; defined as strong staining of 30% or more of the stromal cells). Antibodies against Cav-1, vimentin, and β-actin were purchased from Abcam (Cambridge, MA, USA or Santa Cruz, California, USA).

### LCM

Frozen sections (5 µm thick) of pancreatic cancer, paraneoplastic, and normal tissue were microdissected using a PixCell II LCM system (Arcturus Engineering, Mountain View, California, USA). The laser capture system was equipped with PixCell II image archiving software. The settings of the laser were as follows: spot diameter of 15 µm, pulse duration of 50 ms, and power set to 50 mW. Tissue was microdissected to 10 sections of each sample with a separate “cap” used to capture the material from each section. Typically, 2500 to 3000 laser pulses were used for each cap. After microdissection, the plastic film containing the microdissected cells was removed from the rest of the cap, and all the films containing material from a single sample were placed in a microcentrifuge tube and frozen in liquid nitrogen or stored at −80°C for mRNA extraction. Frozen sections were cut on a cryostat. Sections were thawed and mounted on uncoated glass slides. The frozen sections were fixed in 70% ethanol for 30 s and stained with H&E, followed by three dehydration steps of 5 s each in 70%, 95%, and 99.5% ethanol, and final 5 min dehydration in xylene. Two important factors to ensure satisfactory microdissection and control of selection bias using LCM are: (1) the need for a trained pathologist to discriminate specific populations of diseased cells, such as CAFs, visually; and (2) the absence of cover slip in analyzing microdissected tissue section. The absence of a cover slip and the index matching between the mounting media and the tissue cause the dry tissue section to have a refractile quality that obscures cellular detail at high magnifications. To improve visualization, a drop of xylene is added to the tissue to provide wetting and to facilitate refractive-index matching.

### RT-PCR and Real-time PCR

Total RNA was extracted from pancreatic tumor, paraneoplastic, and normal tissue samples using TRIzol reagent (Gibco BRL, Life Technology, Grand Island, New York, USA).

First-strand cDNA was synthesized from 2 µg of total RNA using the RevertAid Kit (Fermentas MBI, Waltham, Massachusetts, USA). The PCR primer sets were designed as follows: 1) for Cav-1 (140 bp), forward CTGGGCTGTTCTCGCTTCG-3′ and reverse 5′-CTCTCCTCTTCCTTCTCTTCTTCC-3′; and 2) for β-actin (179 bp), forward 5′-ATCGTGCGTGACATTAAGGAGAAG-3′ and reverse 5′-AGGAAGGAAGGCTGGAAGAGTG-3′. PCR conditions included an initial denaturation step for 5 min at 94°C followed by 22 cycles of amplification: 30 sec at 94°C, 30 sec at 55°C, and 30 sec at 72°C. After the last cycle, a final extension was performed at 72°C for 10 min. The housekeeping gene, β-actin, was used as an internal control.

Real-time quantitative PCR was performed with Platinum SYBR Green qPCR SuperMix UDG (Invitrogen, USA) using the Rotor-Gene RG-3000 (Corbett Research, Doncaster Victoria, Australia). For each amplicon, the amount of Cav-1 and β-actin was determined from a standard curve generated by serial dilution. Prior to amplification, the samples were incubated at 95°C for 10 min, and each amplification cycle consisted of denaturation for 45 sec at 95°C, annealing for 30 sec at 57°C, and extension for 30 sec at 73°C. The amount of target genes in the cDNA samples was calculated based on the threshold cycle (Ct). The PCR signals were quantitated by densitometric analysis using Quantity One analysis software.

### Isolation and Culture of Primary Human Fibroblasts

CAFs and normal fibroblasts (NFs) were isolated from pancreatic cancer and non-cancerous partial pancreatectomy specimens, respectively. Specimens were collected and transferred to the laboratory. After several washings with sterile phosphate-buffered saline (PBS), 1 cm^2^ sections of tissues were placed into the wells of culture flasks. Once the tissue appeared to attach to the flasks (5 h to 6 h), Dulbecco’s modified Eagle medium containing 10% fetal bovine serum was added. Specimens were inspected daily for the emergence of fibroblasts, and the medium was changed after 24 h and every third day, thereafter. Tissue samples were removed from the cultures once fibroblasts reached 70% confluence (approximately two weeks), and fibroblasts were transferred to large tissue culture vessels. All fibroblasts used for this study were from passages 3 to 5. Fibroblasts were verified by vimentin positive immunohistochemistry staining.

### Immunofluorescence Assay

Exponentially growing cells were seeded on 25 mm square glass cover slips placed in 35 mm diameter culture dishes. After treatment, the cells were fixed with 4% formaldehyde for 5 min, permeabilized with 0.2% solution of Triton X-100 in PBS, and blocked with 2% bovine serum albumin (BSA)-PBS for 30 min. Slides were incubated with anti-Cav-1 overnight. Fluorescent imaging was obtained with a confocal laser scanning microscope (Carl Zeiss MicroImaging, Inc.).

### Fluorescence in situ Hybridization

HER-2/neu gene was amplified with dual-color (fluorescence in situ hybridization) FISH using a Passvision HER-2 DNA probe kit (Vysis Inc. Downers Grove, Illinois, USA) according to manufacturer’s instructions. Then, 4 µm thick tissue sections were baked overnight at 56°C and were subjected to deparaffinization, enzyme digestion, and fixation. The slides were then denatured in 70% formamide/two-time standard saline citrate at 72°C for 5 min. After buffer wash, 10 µL of a mixture of two directly labeled probes (HER-2/neu specific sequence probe) was added to the tissue sections, and hybridization was performed at 37°C for 14 h to 18 h. The slides were then washed in a post-hybridization wash at 72°C, counterstained with 4, 6-diamidino -2-phenylindole (DAPI), mounted, and stored in the dark before signal enumeration. HER-2/neu-spectrum orange probe contains a DNA sequence specific to the HER-2/neu gene locus and hybridized to the region 17q11.2-q12 of human chromosomes. Chromosome enumeration probe 17 (CEP17)/spectrum green probe containing alpha-satellite DNA that hybridizes to the D17Z1 locus (centromere region of chromosome 17) was used as control. The slides were observed under fluorescence microscope equipped with a digital camera (DP50; Olympus, Tokyo, Japan). For each specimen, gene amplification was scored when a minimum of 20 cancer cell nuclei exhibited a HER-2/CEP17 ratio ≥2 or when a HER-2 signal cluster was observed.

### Blood Sampling and Enrichment of CTCs

Consent forms were approved by the Ethics Review Committee of the Human Subjects Committee of Xi’an Jiaotong University, China and signed by all study patients. First, 7.5 mL samples of peripheral blood were collected in BD Vacutainer tubes (Becton, Dickinson and Company, Franklin, New Jersey, USA) and washed with PBS. To avoid epithelial cell contamination during venipuncture, all samples were collected after discarding the first 2 mL of blood. Red blood cells (RBCs) were mixed with 45 mL of lysis buffer (155 mM NH_4_Cl, 10 mM KHCO_3_, 0.1 mM EDTA), followed by rotation for 8 min and centrifugation (600 g for 5 min) to remove RBCs. The resulting cell pellet was resuspended in PBS and subsequently incubated with 0.5 mL of antileukocyte surface marker CD45 monoclonal antibody-coated magnetic beads for 30 min, followed by separation of magnetic beads using a magnetic stand (Promega, Madison, Wisconsin, USA). Supernatants were transferred into a new tube, and subsequently centrifuged at 800 g for 3 min. Cell pellets were spotted on glass slides, followed with (immunofluorescence in situ hybridization) imFISH staining.

### imFISH Staining (CEP8-CD45-DAPI)

Negative enrichment of tumor cells was performed by using immunomagnetic beads, followed by identification with cytology analysis. FISH was performed using centromere DNA probes of chromosome 8 (yellow) (Vysis Inc. Downers Grove, Illinois, USA), and immunofluorescence assay was performed using anti-CD45 (red) (Santa Cruz, California, USA). The slides were washed thrice with tris-buffered saline (TBS) (10 mM Tris, 2.8 mM KCl, 137 mM NaCl, pH 7.4) containing 0.2% BSA for 3 min and subsequently rinsed with TBS once. Cells were mounted with mounting medium containing the nuclear dye DAPI. A blinded review of the fluorescent images by three technicians confirmed the identity of the CTCs from three-color fluorescent images that were magnified 400 times. Evaluation criteria for CTC identification from fluorescent images included both CEP8≥3 and CD45 (−) staining pattern overlying the DAPI staining of the nucleus.

### Statistical Analysis and Patient Outcome

Data were analyzed by using the chi-square test (χ2) or two-sided Fisher exact test, as appropriate. Pearson correlation coefficient was used to measure the strength of the association among Cav-1, enumeration of CTCs, and HER-2/neu expression levels. Survival rate was calculated by using the Kaplan–Meier method, and differences were examined by the log-rank test. Factors found to be significant were then selected for a stepwise Cox’s multivariate proportional hazard model to determine their prognostic values. *P*<0.05 was considered statistically significant. All statistical analyses were performed using SPSS Version 13.0 (SPSS, Chicago, Illinois, USA).

## Results

### Expression of Stromal Cav-1 in Pancreatic Cancer

**Figure 1:**
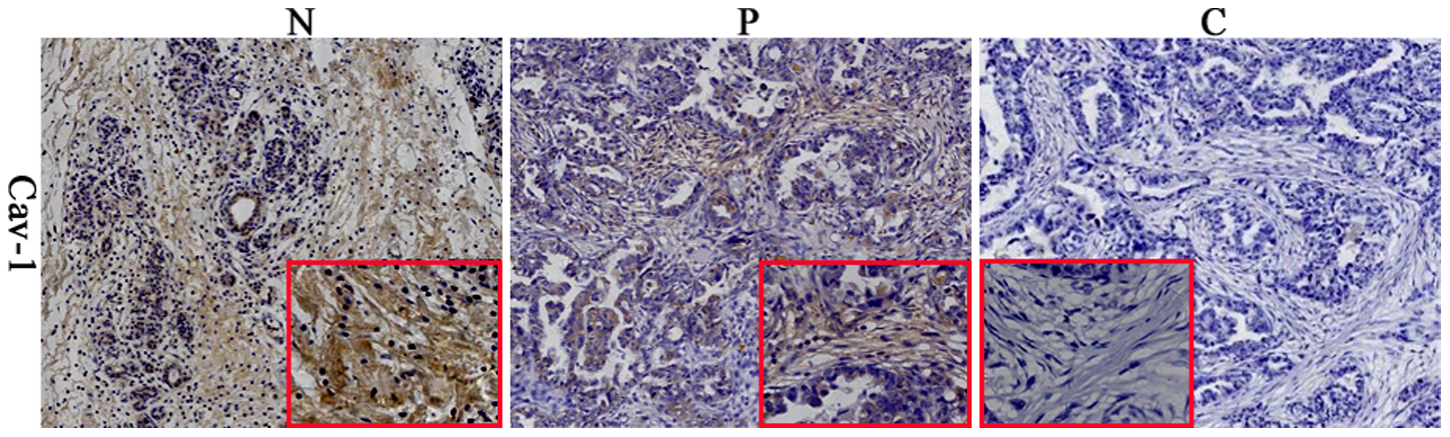
Representative immunohistochemistry results of stromal Cav-1 expression in pancreatic tissue. Paraffin sections were immunostained as described in the methods section. (N) Normal tissue strongly positive for Cav-1 expression; (P) Paracancer tissue moderate for Cav-1 expression; (C) Tumor tissue with negative Cav-1 expression (×400).

Cav-1 is expressed both on the membrane and in the cytoplasm of cells [16]. To examine the expression status of stromal Cav-1 in pancreatic cancer, we used immunohistochemistry to evaluate the pancreatic cancer, paraneoplastic, and normal tissue sections. Our results showed representative images of Cav-1 antibody stained tissue sections (Fig. 1), thus highlighting the differences observed in Cav-1 immunostaining in the fibroblastic stromal compartment. Interestingly, Cav-1 expression was strong and confined mainly to the stroma in paraneoplastic and normal tissues but was only detected occasionally in tumor stroma. In 45 cancer specimens, 6 (13.3%) patients showed high levels of stromal Cav-1 staining, whereas 8 (17.8%) showed a lower intermediate level of staining, and 31 (68.9%) showed an absence of stromal Cav-1 staining.

### PCR Analyze in Laser Captured Samples

**Figure 2:**
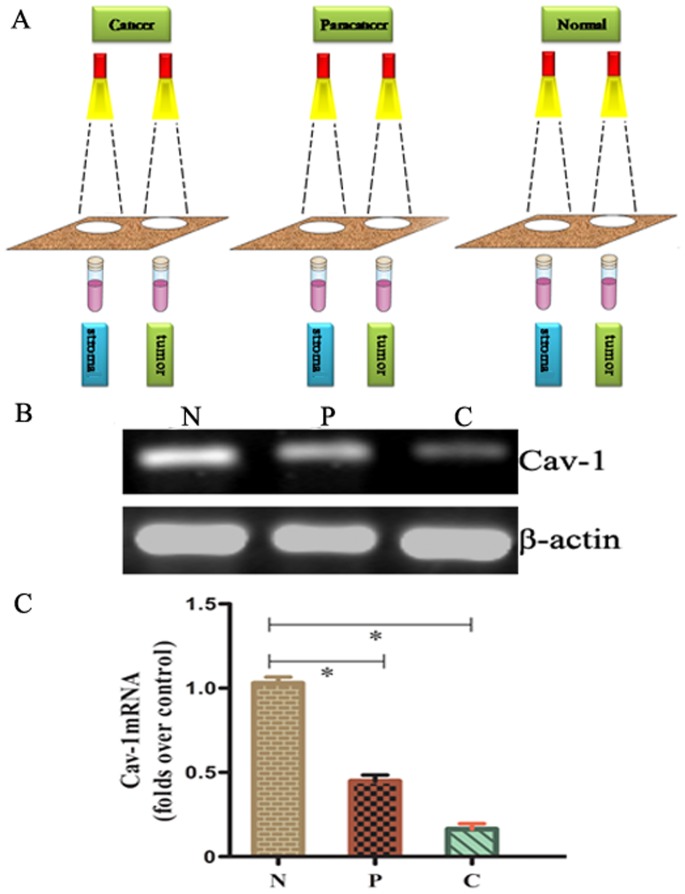
The mRNA expression of stromal Cav-1 in laser-captured samples by RT-PCR and Real-time PCR. **A:** Schematic diagram representing the LCM technique. **B:** mRNA expression of stromal Cav-1 in laser-captured samples as determined by RT-PCR. **C:** Quantification of mRNA by real-time quantitative PCR. Data from at least three independent experiments with duplicate determinations are expressed as means ± SEM. (N) Normal tissue; (P) Paracancer tissue; (C) Tumor tissue. *P<0.05 was considered statistically significant.

Loss of stromal Cav-1 is a single independent predictor of early breast cancer recurrence and progression [17]. Our immunohistochemical data also showed lower stromal Cav-1 expression in pancreatic cancer. To verify the immunohistochemical data further, we separated and purified stroma using LCM. The stroma cells of pancreatic specimens were microdissected successfully from the tumor, paraneoplastic, and normal sections. The LCM technique caused no visible alteration in the morphology of the dissected specimens (Fig. 2A). We sequentially analyzed the mRNA expression of Cav-1 in laser-captured stroma samples by RT-PCR and Real-time PCR (qPCR). The mRNA level of Cav-1 was significantly degraded in laser-captured tumor stroma samples compared with the paraneoplastic and normal tissue (Figs. 2B, 2C) (*P*<0.05).

### Expression of Cav-1 in CAFs, PAFs, and NFs

**Figure 3:**
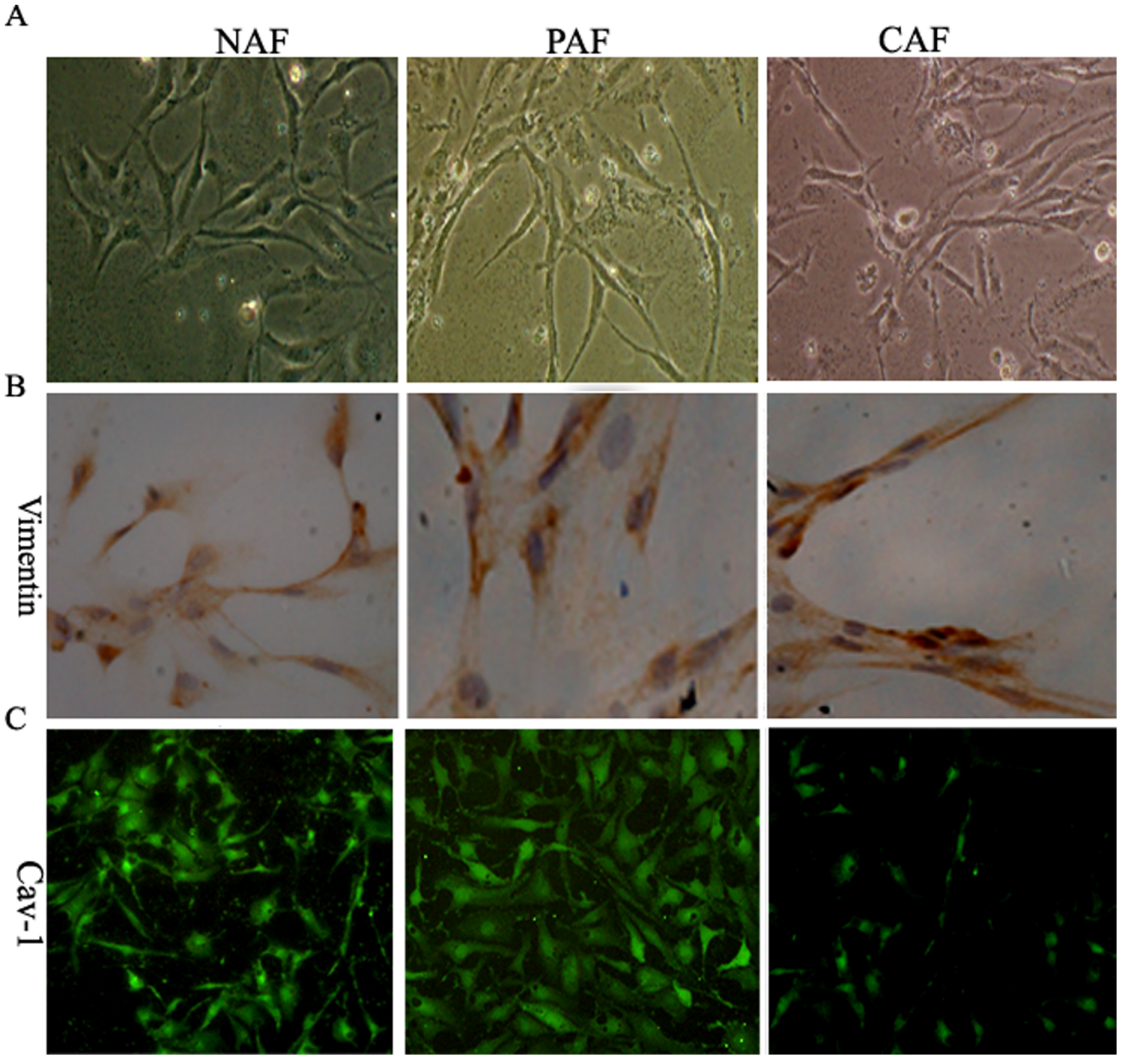
Immunodetection of Cav-1 protien in CAFs, PAFs, and NFs. **A:** Cell morphological characteristic of CAFs, PAFs, and NFs (×100). Extracted CAFs from pancreatic cancer and PAFs and NFs from paracancerous tissue and non-cancerous partial pancreatectomy specimens. **B:** Different vimentin expression levels in stroma CAFs, PAFs, and NFs were assessed by immunohistochemistry (×200). **C:** Cav-1 protein expression in CAFs and NFs stained with FITC labeling-IgG antibody and analyzed by confocal microscopy. Cav-1 fluorescence signal in CAF is lower than in PAFs and NFs (×200). (CAFs) Cancer associated fibroblasts; (PAFs) Paracancerous associated fibroblasts; (NFs) Normal associated fibroblasts.

To understand further the stromal Cav-1 protein expression in pancreatic stroma (mostly fibrocytes), we first extracted CAFs from pancreatic cancer cells, paracancer-associated fibroblasts (PAFs), paracancerous tissue, and NFs from non-cancerous partial pancreatectomy specimens. Different vimentin expression levels in stroma CAFs, PAFs, and NFs were assessed by immunohistochemistry analysis. These results also verified that our cell extraction was successful (Figs. 3A, 3B). Subsequently, we determined and analyzed stromal Cav-1 expression in CAFs and NFs stained with fluorescein isothiocyanate labeling-IgG antibody by confocal microscopy. Cav-1 fluorescence signal in CAFs was lower than that in PAF and NF specimens (Fig. 3C), which suggests that Cav-1 is lost in a pancreatic cancer microenvironment.

### Relationships between Stromal Cav-1 Expression and Clinicopathological Parameters

Table 1 summarizes the associations of stromal Cav-1 protein expression and clinicopathological parameters in pancreatic cancer. The stromal Cav-1 loss was seen in six of 14 stage I and II cases (42.9%) and was significantly lower than in stage III (77.8%, 21 of 27 cases) and stage IV cases (100.0%, 4 of 4 cases) (*P = *0.018). Stromal Cav-1 loss was also associated with lymph node metastasis (*P* = 0.014) and distant metastasis (*P* = 0.027). We also investigated the relationship of age, sex, histological grade, and tumor size with stromal Cav-1 expression. However, statistically significant relationships were not observed (*P*>0.05).

**Table 1:** Association between stromal Cav-1 expression and clinicopathologic factors in pancreatic cancers.

		Cav-1			
Variables	Case	0	1	2	*P* value
	N = 45	31 (%)	8 (%)	6 (%)	
*Age (years)*					0.637
>60	28	20 (71.4)	5 (17.9)	3 (10.7)	
≤60	17	11 (64.7)	3 (17.6)	3 (17.6)	
*Sex*					0.763
Female	21	14 (66.7)	4 (19.0)	3 (14.3)	
Male	24	17 (70.8)	4 (16.7)	3 (12.5)	
*Histological grade*					0.290
I	7	3 (42.9)	2 (28.6)	2 (28.6)	
II	20	15 (75.0)	2 (10.0)	3 (15.0)	
III	18	13 (72.2)	4 (22.2)	1 (5.6)	
*Tumor size*					0.779
≤2 cm	15	9 (60.0)	5 (33.3)	1 (6.7)	
2–5 cm	26	19 (73.0)	2 (7.6)	5 (19.2)	
>5 cm	4	3 (75.0)	1 (25.0)	0 (0.0)	
*Lymph node metastasis*					0.014
Negative	17	8 (47.1)	4 (23.5)	5 (29.4)*	
Positive	28	23 (82.1)	4 (14.3)	1 (3.6)*	
*Distant metastasis*					0.027
Negative	39	26 (66.7)	7 (17.9)	6 (15.4)*	
Positive	6	5 (83.3)	1 (16.7)	0 (0.0)*	
*TNM stage*					0.018
I/II	14	6 (42.9)	2 (14.3)	6 (42.9)*	
III	27	21 (77.8)	6 (22.2)	0 (0.0)	
IV	4	4 (100.0)	0 (0.0)	0 (0.0)*	

Note: Cav-1 staining was scored (0; no staining) as negative, merge scored (1; weak) and (2; strong) as positive, then compared this two groups using χ^2^ test. * *P*<0.05.

### Loss of Stromal Cav-1 Correlates with HER-2/neu Gene Amplification

**Figure 4:**
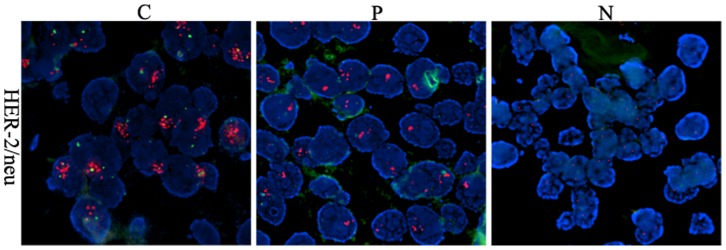
Representative photomicrograph of HER-2/neu gene amplification in pancreatic cancer. (N) Normal tissue negative for HER-2/neu gene amplification; (P) Paracancer tissue moderate for HER-2/neu gene amplification; (C) Tumor tissue positive for HER-2/neu gene amplification (×1000).

HER-2/neu is a transmembrane growth factor receptor, the overexpression of which is recognized as an independent adverse prognostic factor in several cancers, including pancreatic cancer [18], [19], [20], [21], [22], [23], [24], [25]. To explore further whether stromal Cav-1 loss is an adverse prognostic biomarker in pancreatic cancer, the correlation between stromal Cav-1 expression and HER-2/neu gene amplification was analyzed. HER-2/neu gene amplification was evaluated using FISH, which is considered the gold standard for measuring the percentage of positive cells. Our results showed that 64.4% of pancreatic adenocarcinomas exhibited HER-2/neu gene amplification (Fig. 4). Stromal Cav-1 loss had a positive correlation with HER-2/neu gene amplification (r = −0.697, *P* = 0.000; Table 2). Fourfold stromal Cav-1 loss was observed in samples displaying HER-2/neu gene amplification. By contrast, in peritumoral and normal samples, stromal Cav-1 expression was high when HER-2/neu gene was not amplified. In conclusion, stromal Cav-1 loss was positively correlated with the amplification of the HER-2/neu gene.

**Table 2:** Relationship between expression of stromal Cav-1 and HER-2/neu amplification.

		Cav-1			
Gene	Case	0	1	2	*P* value
	N = 45	31(%)	8(%)	6(%)	
*HER-2/neu*					0.007
Amplification	29	24 (82.8)	3 (10.3)	2 (6.9)*	
Normal	16	7 (43.8)	5 (31.2)	4 (25.0)*	

Note: Cav-1 staining was scored (0; no staining) as negative, merge scored (1; weak) and (2; strong) as positive, then compared this two groups using χ^2^ test.

*P<0.05.

### Loss of Stromal Cav-1 Correlates with CTC Enumeration

**Figure 5:**
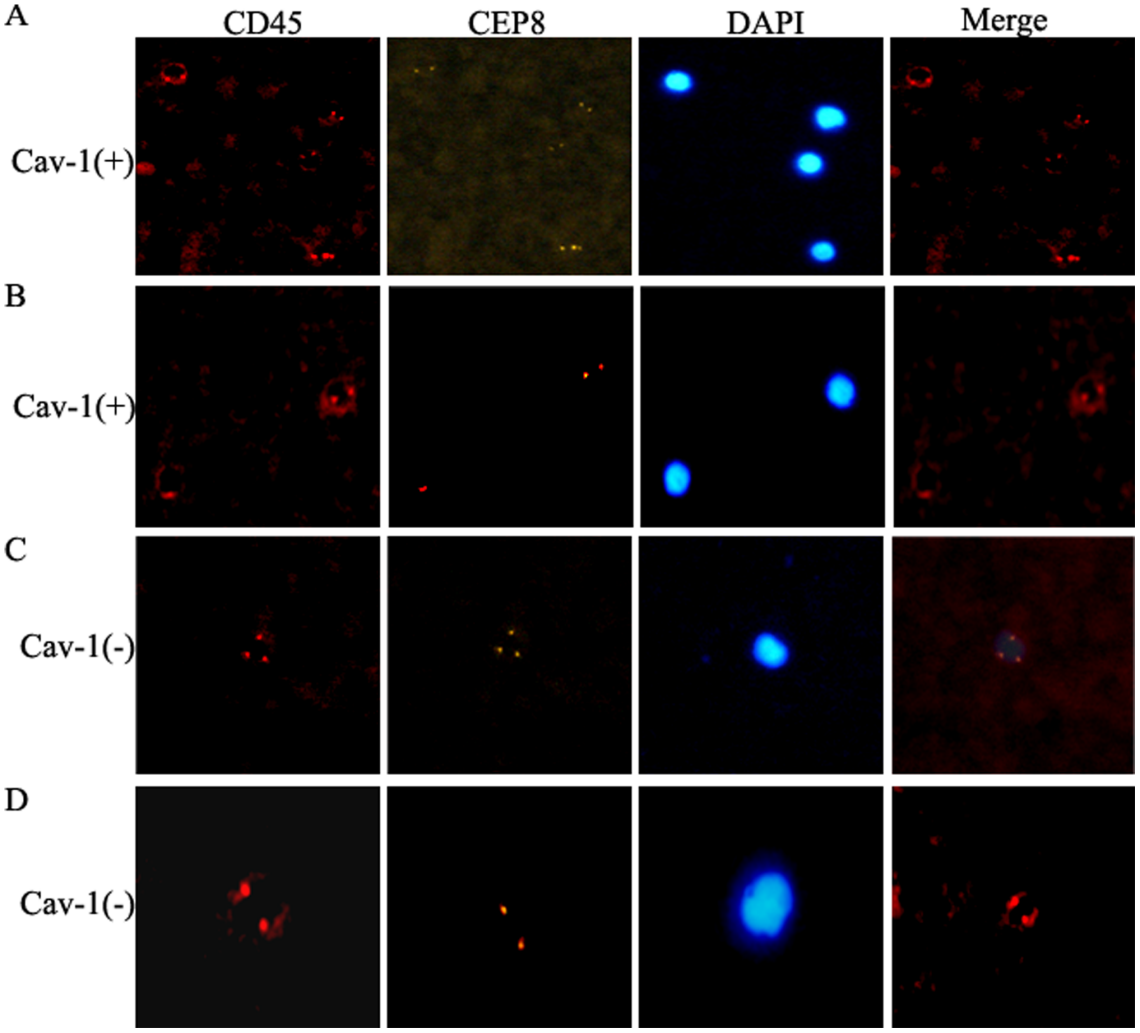
CTCs stained with anti-CD45-CEP8-DAPI in peripheral blood of pancreatic cancer patients. **A:** CTCs negative in patients with stromal Cav-1 loss. **B:** CTCs positive in patients with stromal Cav-1 loss. **C:** CTCs negative in patients with stromal Cav-1 expression. **D:** CTCs positive in patients with stromal Cav-1 expression. The white scale bar indicates 10 µm (x400).

CTCs are tumor cells shed from the primary tumor into the circulating blood. The presence of CTCs in the peripheral blood of patients has been associated with metastasis and poor survival, although the biologic significance of CTCs attributed to tumor genomic instability and potential metastatic inefficiency has been debated [26]. Based on its clinical relevance, CTC is recommended by the American Society of Clinical Oncology to be an acceptable cancer marker [27]. To explore further whether stromal Cav-1 loss is an adverse prognostic biomarker, the correlation between stromal Cav-1 expression and CTC enumeration was analyzed. Our results showed that CTCs were detected in 35.71% (5/14) of pancreatic cancer patients with stromal Cav-1 expression versus 77.42% (24/31) of patients with stromal Cav-1 loss (Fig. 5). Moreover, significantly higher CTC enumeration was observed in patients with stromal Cav-1 loss than in patients with stromal Cav-1 expression (18.5±2.0 in 7.5 mL of blood vs 6.0±1.5 in 7.5 mL of blood). In conclusion, stromal Cav-1 loss was positively correlated with CTC enumeration.

### Prognostic Values of Loss of Stromal Cav-1 in Patients with Pancreatic Cancer

**Figure 6:**
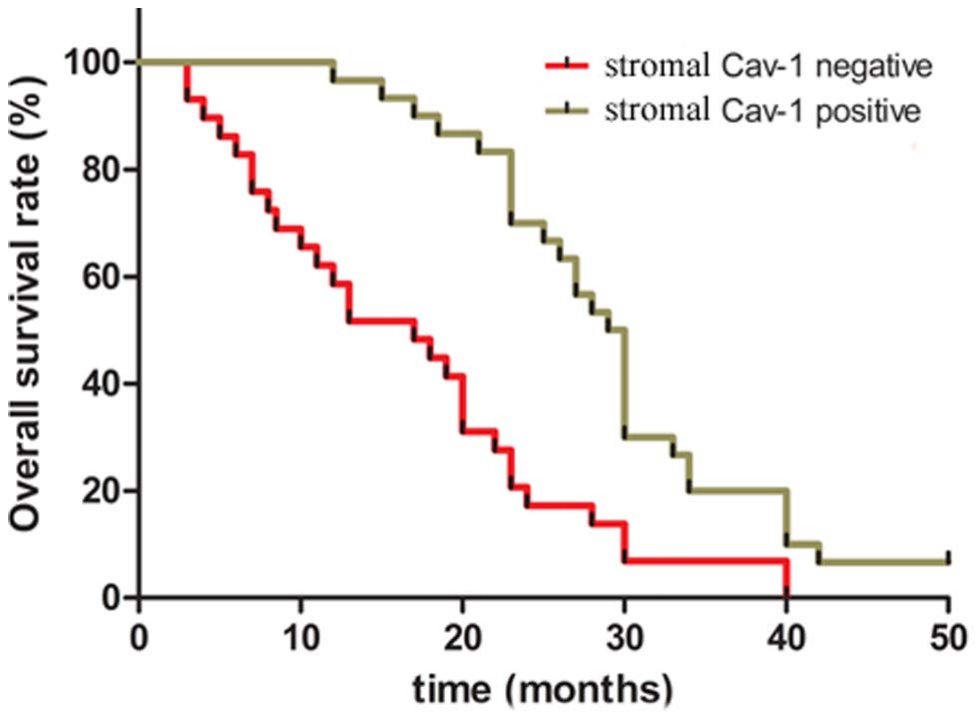
Kaplan–Meier analysis of the overall postoperative survival curves in pancreatic cancer cases according to immunohistochemical staining as positive or negative of stromal Cav-1 expression.

To determine the prognostic value of stromal Cav-1 for pancreatic cancer, we analyzed the cumulative survival of patients according to their Cav-1 status (Fig. 6). Stromal Cav-1 absence, as indicated by the lack of staining, was regarded as negative, whereas weak or strong staining was regarded as positive. The cumulative survival rate in stromal Cav-1 negative patients (n = 31) at three years was 8.8% (median survival time of 16 months). By contrast, the cumulative survival rate in stromal Cav-1 positive patients (n = 14) was 20.2% (median survival time of 28 months), a difference that was statistically significant (*P*<0.05).

Based on multivariate analysis, the summary OR of lymph node metastasis on cumulative survival times at three years was 1.32 (95% CI, 1.10–1.63), and TNM (III+IV/II+I) stage (OR = 1.27, 95% CI, 1.15–1.46) was an independent prognostic factor for overall survival time in study patients with pancreatic cancer. The loss of stromal Cav-1 protein was also an independent prognostic factor for overall survival time (*P = *0.006). However, tumor diameter and other clinical parameters were dependent prognostic factors.

## Discussion

Solid tumors are no longer considered simply as clonal expansions of cancer cells. In addition to acquired cell intrinsic properties, tumor initiation and growth are supported by an abundance of parenchymal, inflammatory, and stromal cell types, which infiltrate and surround the tumor [28]. Our findings highlight the importance of an evolving microenvironment and suggest that therapy should target both neoplastic cells and supportive stromal cells [29]. The autophagic tumor stroma model of cancer metabolism [30] suggests that the loss of stromal Cav-1 as a key regulator is a potential therapy target, further suggesting that stromal Cav-1 expression in stromal cells can be of prognostic significance. Moreover, a loss of stromal Cav-1 has been reported to be a predictor of early tumor recurrence, lymph node metastasis, tamoxifen resistance, and poor clinical outcome in human breast cancer patients [17]. We investigated stromal Cav-1 expression in pancreatic cancer to evaluate a potential role for stromal Cav-1 as a prognostic marker. Stromal Cav-1 was downregulated in pancreatic cancer compared with paraneoplastic and normal tissue. Loss of stromal Cav-1 is closely correlated with advanced TNM stage, lymph node metastasis, distant metastasis, and poor prognosis. The loss of stromal Cav-1 was correlated with the amplification of classic markers for tumor progression (HER-2/neu gene). More importantly, the loss of stromal Cav-1 was associated with metastasis because circulating tumor cells were found in patient blood. These findings extend our understanding of the function of stromal Cav-1 as a diagnostic marker. Most importantly, to our knowledge, this study is the first to show that loss of stromal Cav-1 in pancreatic cancer is a negative prognostic indicator. However, the work of Witkiewicz et al. [31] revealed that the co-expression of FASN and Cav-1 may be an informative clinical marker. Witkiewicz et al. found that Cav-1 and FASN had higher expressions in PDAs than in PanIN. In addition to staining neoplastic epithelial cells, Cav-1 was also present in fibroblasts of the desmoplastic cancer stroma. Stromal cells in normal pancreas or chronic pancreatitis tissue adjacent to tumor cells were negative, which is in contrast to our findings. This difference may be attributed to the possibility that the study by Witkiewicz et al. occurred during the immunolocalization of the two molecules (FASN and Cav-1), and the results may be attributed to mutual interference. Moreover, the used Cav-1 Ab had a different specificity. The method used by Witkiewicz et al. (immunohistochemistry) is different from that used in this study, in which PCR was employed with respect to quantification. Finally, sample heterogeneity may have also affected the results. Although the findings of Witkiewicz et al. [17] are consistent with those of our study, further study should be conducted on this topic.

CTCs are tumor cells shed from the primary tumor into blood circulation. The presence of CTCs in the peripheral blood of patients has long been associated with metastasis and poor survival [32] and is now considered an acceptable cancer marker [27]. However, current techniques are limited. The only commercially available CTC test (Cell Search; Veridex LLC, North Raritan, New Jersey, USA) has a detection rate of 50% in late-stage patients [33]. In this study, we detect circulating tumor cells harboring negative enrichment by using the immunomagnetic bead method, followed by identification with cytology analysis, immunofluorescence, and imFISH. Detection rates increased to 77.42% in patients with Cav-1 loss, which suggests that imFISH staining can be used as a detection method for CTCs in future studies. Loss of stromal Cav-1 is associated with the possibility of metastasis compared with higher stromal Cav-1 expression.

Molecular analysis of tumor tissue requires the isolation of specific populations of cells. The presence of contaminated cells within a sample remains a major obstacle to meaningful biological analysis. LCM is a recently developed technique that enables the rapid and reliable procurement of a specific type of cell from a tissue section in one step, under direct microscopic visualization [34]. LCM has been used to isolate specific types of cells both for DNA and RNA analysis. This technique has been applied to the study of prostate cancer, breast cancer, and ovarian cancer [35], [36]. In this study, we investigated the feasibility of using cells obtained by LCM for stromal Cav-1 analysis. Stroma cells of pancreatic specimens were microdissected successfully from tumor and normal tissue sections. LMC caused no visible alteration in the morphology of the dissected specimens. We sequentially analyzed the mRNA expression in laser-captured stroma samples that were similarly downregulated.

Cav-1, a 21–24 kD protein, is a component of caveolae invaginated microdomains of the plasma membrane that is present in most mammalian cells [37]. Cav-1 was described as the main structural protein in caveolae and was believed to be a key molecule involved in oncogenic transformation and malignant progression [38]. Cav-1 serves an important regulatory function in several signaling pathways in cellular transformation, including those mediated by the Src family of tyrosine kinases, epidermal growth factor receptor, HER-2/neu, protein kinase C, Wnt, and Erk1/2 [6], [39], [40], [41]. Cav-1 has been suggested to act either as a tumor suppressor or as an oncogene, depending on the tumor type and/or tumor stage. These various effects may be explained by the activation status of different domains of Cav-1 or the expression levels of other molecules that interact with Cav-1 in these different signaling pathways [42]. Several studies have recently assessed the potential function of Cav-1 in tumor progression [8], [43], [44]. However, the relationship between Cav-1 expression and cancer progression remains unclear at both the cellular and molecular levels. Owing to the autophagic tumor stroma model of cancer metabolism, the loss of Cav-1 protein likely affects tumor progression via metabolism transform, which is worthy of further study.

In summary, stromal Cav-1 is downreguated in pancreatic cancer and is closely related to advance TNM stage, lymph node metastasis, and poor prognosis. Stromal Cav-1 loss is highly correlated with conventional tumor markers and HER-2/neu amplification. Therefore, the loss of stromal Cav-1 may be used as a novel biomarker for pancreatic cancer aggressiveness in a select panel of biomarkers.
